# Aberrant Protein Phosphorylation in Cancer by Using Raman Biomarkers

**DOI:** 10.3390/cancers11122017

**Published:** 2019-12-13

**Authors:** Halina Abramczyk, Anna Imiela, Beata Brożek-Płuska, Monika Kopeć, Jakub Surmacki, Agnieszka Śliwińska

**Affiliations:** 1 Laboratory of Laser Molecular Spectroscopy, Institute of Applied Radiation Chemistry, Faculty of Chemistry, Lodz University of Technology, Wroblewskiego 15, 93-590 Lodz, Poland; anna.imiela@p.lodz.pl (A.I.); beata.brozek-pluska@p.lodz.pl (B.B.-P.); monika.kopec@p.lodz.pl (M.K.); jakub.surmacki@p.lodz.pl (J.S.); 2 Faculty of Medicine, Medical University of Lodz, Chair of Department of Nucleic Acids Biochemistry, Pomorska 251, 92-213 Lodz, Poland; agnieszka.sliwinska@umed.lodz.pl

**Keywords:** tyrosine phosphorylation, Raman spectroscopy, Raman imaging, cancer, biomarker

## Abstract

(1) Background: Novel methods are required for analysing post-translational modifications of protein phosphorylation by visualizing biochemical landscapes of proteins in human normal and cancerous tissues and cells. (2) Methods: A label-free Raman method is presented for detecting spectral changes that arise in proteins due to phosphorylation in the tissue of human breasts, small intestines, and brain tumours, as well as in the normal human astrocytes and primary glioblastoma U-87 MG cell lines. Raman spectroscopy and Raman imaging are effective tools for monitoring and analysing the vibrations of functional groups involved in aberrant phosphorylation in cancer without any phosphorecognition of tag molecules. (3) Results: Our results based on 35 fresh human cancer and normal tissues prove that the aberrant tyrosine phosphorylation monitored by the unique spectral signatures of Raman vibrations is a universal characteristic in the metabolic regulation in different types of cancers. Overexpressed tyrosine phosphorylation in the human breast, small intestine and brain tissues and in the human primary glioblastoma U-87 MG cell line was monitored by using Raman biomarkers. (4) We showed that the bands at 1586 cm^−1^ and 829 cm^−1^, corresponding to phosphorylated tyrosine, play a pivotal role as a Raman biomarker of the phosphorylation status in aggressive cancers. We found that the best Raman biomarker of phosphorylation is the 1586/829 ratio showing the statistical significance at *p* Values of ≤ 0.05. (5) Conclusions: Raman spectroscopy and imaging have the potential to be used as screening functional assays to detect phosphorylated target proteins and will help researchers to understand the role of phosphorylation in cellular processes and cancer progression. The abnormal and excessive high level of tyrosine phosphorylation in cancer samples compared with normal samples was found in the cancerous human tissue of breasts, small intestines and brain tumours, as well as in the mitochondria and lipid droplets of the glioblastoma U-87 MG cell line. Detailed insights are presented into the intracellular oncogenic metabolic pathways mediated by phosphorylated tyrosine.

## 1. Introduction

For many years, cancer research has been dominated by the genomic approach, in which variations in the sequences of certain genes, oncogenes and tumour suppressor genes are the main causes of the disease. The study of cancer genomes has improved our understanding of the mechanisms of cancer development and has led to new methods for cancer diagnosis and treatment. Despite the search for universal behaviours among cancer types, TCGA (The Cancer Genome Atlas Network, 2012) revealed the abnormalities of many types of cancer, for example, finding over 30,000 mutations in breast cancer cells. Thus, the available evidence appears to suggest that scientific research based solely on cancer genomics cannot continue.

Presently, a growing number of reports have led to increased interest in the benefits of metabolic regulation in cancers. While the transformation of oncogenic genes can be directly connected to cancer cell metabolism, mutated metabolic enzymes may also trigger malignant transformations [1,2,3].

Understanding the mechanism of the altered energy metabolism that replaces the more efficient oxidative phosphorylation to yield adenosine triphosphate (ATP) by the less efficient process of glycolysis represents the pivotal problem that must be solved to advance the study of cancer. During the past decade, many studies have suggested that the metabolic fate of pyruvate is regulated by tyrosine phosphorylation [4,5,6].

Tyrosine phosphorylation has been suggested to be involved in metabolic enzymes, such as the M2 isoform of pyruvate kinase (PKM2), which contributes to the shift from the highly active tetrameric form to the less active dimeric form, thereby reducing the glycolytic flux. It has also been suggested that phosphorylation of PKM2 alone is unlikely to be sufficient to induce the glycolytic switch [5,7]. It was shown that phosphorylation of other enzymes, such as pyruvate dehydrogenase kinase 1 (PDHK1, also known as PDK1), by tyrosine kinases is involved in blocking of the mitochondrial pyruvate metabolism in cancer cells [7]. Other studies have suggested that the increased uptake of glutamine and its conversion into pyruvate can contribute to the increased levels of lactate produced by cancer cells [8].

Various experimental procedures have been used for metabolism monitoring, such as immunofluorescence microscopy, mitochondrial subfractionation assays, mitochondrial pyruvate consumption [1] and [1-14C]-pyruvate conversion assays [7]. Some cell fractionation kits allow the rapid and simple preparation of mitochondrial-, cytoplasmic- and nuclear-containing fractions from cultured cells. Kits for analysing phosphorylation targets are also available. Current platforms for proteomic analysis, such as immunoassays (e.g., western-blot, ELISA, immunohistochemistry and planar and bead arrays) using fluorescent or radio-labelled probes [9,10,11] mass spectrometry [11,12,13] flow cytometry [14] and protein sequencing [11] can detect aberrant changes in the phosphorylated protein levels.

However, there are some limitations to the potential use of these methods for clinical purposes due to the immunohistochemical artefacts and cross-reactivity between the different epitopes [15,16] and the time-consuming procedures with specific radioactive or fluorescent tags [17]. These methods are also characterized by the limited sensitivity, specificity and spatial or spectral resolution [18]. The design and development of biosensors are extremely laborious and usually requires the transfection and expression of biosensor proteins within the cell, which is incompatible with the clinical settings. Therefore, the development of techniques for simple and fast analysis of the phosphorylation status of a purified protein that can be implemented in clinical settings remains a challenging endeavour. These limitations have led to the search for new methods of diagnosing and monitoring the metabolism in disease development. Analyses of the extracted components from the cells or tissues.

In recent years, literature reports from around the world clearly indicate that molecular spectroscopy, particularly Raman spectroscopy and Raman imaging, will play a particularly important role in the possible development of innovative techniques for ‘omics’ research (proteomic, lipidomic, glycomic and epigenetic) and medical diagnostics; these techniques can lead to a revolution in cancer research by providing detailed information about the spatial location of biochemical components in organelles [19,20].

While we are only beginning to understand the epigenetic, proteomic, lipidomic, glycomic and metabolic factors involved in the development of cancer, it has become evident that cancer cells have many “omics” differences compared to normal cells in the same patient. Here, we concentrate on the metabolic reprogramming that is now recognized to be a common feature of many cancer cells. While the list of all possible modifications is not yet complete, the manipulation of the metabolic landscape will become a key element of cancer therapy in the future.

**Figure 1:**
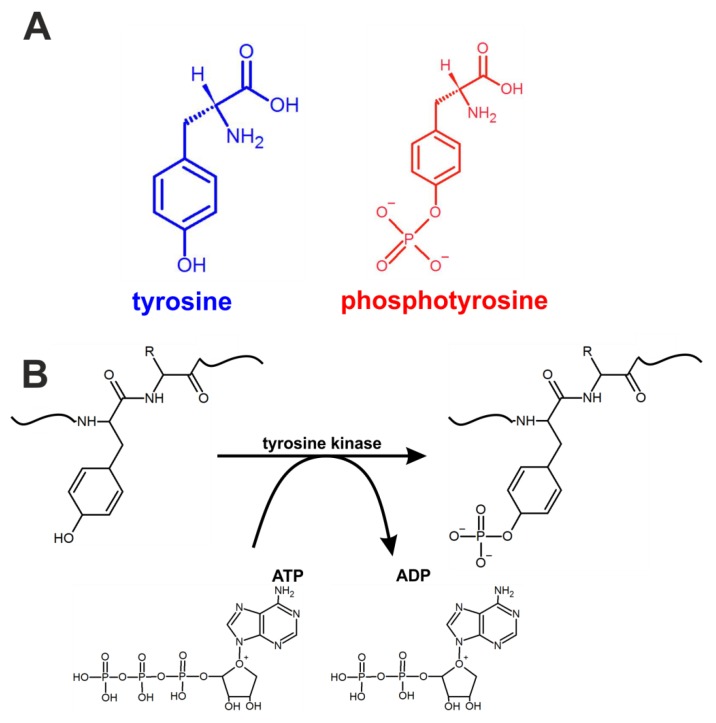
Structural formulae of tyrosine and phosphotyrosine (**A**) and the mechanism of tyrosine residue phosphorylation (**B**) in proteins.

Tyrosine phosphorylation is one of the most important post-translational modifications in cancer cells (Figure 1). Monitoring these modifications at the genomic level is very complicated because approximately 10,000 proteins encoded by the human genome contain covalently bound phosphate. For proteomic research, the situation is slightly simpler because the typical protein kinase can add phosphates to 20 different proteins [21].

Tyrosine kinases belong to two main families, namely nonreceptor tyrosine kinase and receptor tyrosine kinases. The non-receptor tyrosine kinases are located in the cytoplasm, and the receptor tyrosine kinases (RTK) are associated with the cell-surface receptors containing a transmembrane domain and exhibiting activity at the cytoplasmic region [22]. One of the most important receptor families of tyrosine kinases is the ErbB (epidermal growth factor) protein family, which is a subgroup of four structurally related receptor tyrosine kinases: ErbB-1 (epidermal growth factor receptor (EGFR)), ErbB-2 (HER2, human epidermal growth factor receptor 2), ErbB-3 (HER3), and ErbB-4 (HER4). Overexpressed activity of the epidermal growth factor receptor (EGFR) family has been found in many human cancers [23,24]. Phosphorylated tyrosine residues serve as the binding sites for intracellular signal activators (e.g., Ras). The Ras-Raf-MAPK (Human Rat Sarcoma - Rapidly Accelerated Fibrosarcoma – Mitogen Activated Protein Kinase) pathway is a dominant signalling route for the ErbB family that leads to enhanced cell proliferation and inhibition of apoptosis [23]. Oversignalling of ErbB is associated with the development of a broad variety of types of solid tumours. ErbB-1 and ErbB-2 can be found in a variety of human cancers and their excessive signaling may be a crucial factor in the development and malignancy of these tumours [24].

The pivotal role of protein phosphorylation in cancer therapy has been recognized, with approximately thirty percent of the drug discovery programs and R&D (Research and Development) investment conducted by the pharmaceutical industry concentrating on protein kinases and protein phosphatases regulating protein phosphorylation. It has been reported that up to sixty kinase inhibitors have undergone clinical evaluation against cancers, inflammation, diabetes and neurodegenerative diseases [21]. For example, tyrosine kinase inhibitors (TKI) that are capable of restricting the activity of oncogenic phosphorylated kinase proteins have demonstrated long-term improvements in lung cancer survival and multiple TKI therapies are currently approved or undergoing clinical trials [25,26].

In this paper, we concentrate on tyrosine phosphorylation in cancer cells and tissues monitored by Raman spectroscopy and Raman imaging. Compared to the current methods of conventional biology, a Raman-based approach is an interesting alternative for noninvasive monitoring of cellular metabolic processes. Since Raman methods are label-free and do not require any staining or antibody attachment for protein detection, they offer straightforward sample handling that is superior to the complex assays that must consider the sensitivity and specificity to the antibodies. The main advantage of Raman imaging is that in contrast to conventional methods (LC/MS (Liquid Chromatography/Mass Spectrometry), NMR (Nuclear Magnetic Resonance), HPLC (High Performance Liquid Chromatography)) that rely on bulk or fractionated analyses of extracted components, it provides spatial information about the various chemical constituents in defined cellular organelles. Raman-guided analysis does not require the cells to be opened to release the cellular structures.

In this work, we monitor tyrosine phosphorylation by Raman imaging in breast, small intestine and brain cancer cells to assess the diagnostic potential of tyrosine phosphorylation monitoring of cancer cells based on the vibrational signatures obtained by Raman-based methods.

An integrated approach combining results for various types of cancers may offer a broader view of the role of the protein tyrosine kinases in metabolic pathways in cancer development.

## 2. Results

First, we focused on the Raman spectral changes that arise in tyrosine upon phosphorylation. The structural formulae of tyrosine and phosphotyrosine are presented in Figure 1A. A diagram of tyrosine phosphorylation is shown in Figure 1B, where enzyme-catalysed (tyrosine kinase) proton transfer from the (–OH) group on tyrosine residue of the protein stimulates the nucleophilic attack of the terminal phosphate group PO_3_^2-^ on ATP, resulting in the transfer of the phosphate group to tyrosine to form phosphotyrosine and ADP.

**Figure 2:**
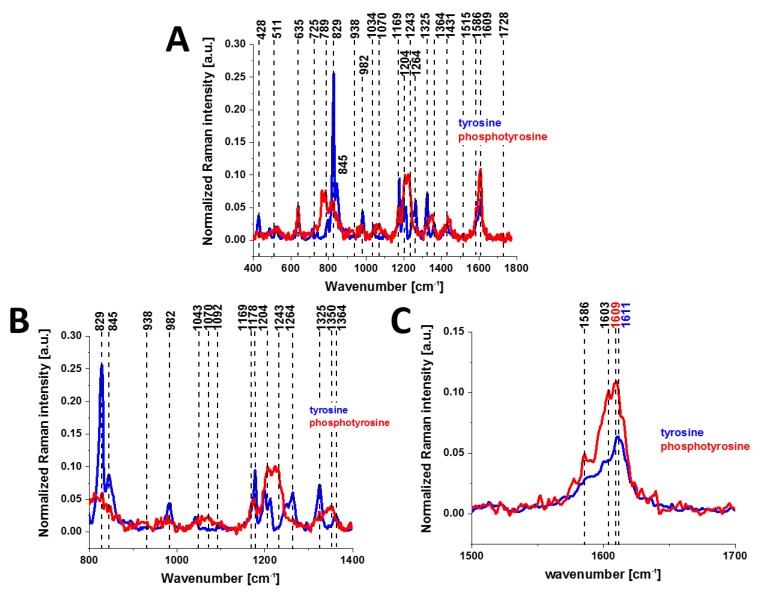
Raman spectra of tyrosine (blue line) and phosphotyrosine (red line) in the spectral region: 400–1800 cm^−1^ (**A**), 800–1400 cm^−1^ (**B**) and 1500–1700 cm^−1^ (**C**); film from a solution, concentration of the solution *c* = 10^−2^ M.

The protonated and phosphorylated forms can be monitored by using Raman scattering because this method can detect the spectral changes in phosphorylated proteins arising from either the phosphate stretching or amide vibrational modes. Figure 2 shows the Raman spectra of tyrosine and phosphorylated tyrosine.

A detailed examination of Figure 2 shows that tyrosine phosphorylation introduces appreciable changes in the Raman spectrum: (1) phosphorylated tyrosine exhibits an additional Raman peak at 1586 cm^−1^ close to the main peak at 1609 cm^−1^ that corresponds to the ring-O stretching mode [27]; (2) the peak of phosphorylated tyrosine at 1609 cm^−1^ is shifted with respect to that of tyrosine observed at 1611 cm^−1^; (3) the characteristic doublet (829 cm^−1^, 845 cm^−1^) of tyrosine corresponding to a Fermi resonance between the first overtone of the aromatic out-of-plane ring bend and the aromatic ring breathing fundamental [28] collapses upon tyrosine phosphorylation with a significant intensity decrease; (4) the band at 1264 cm^−1^ corresponding to Amide III is shifted to 1243 cm^−1^ upon phosphorylation [29,30].

The band positions of aromatic amino acids are sensitive to the microenvironment and may shift by up to 5 cm^−1^ in the Raman spectra of proteins [31]. Vibrations of the PO_4_^−^ phosphate group of phosphorylated tyrosine are observed at 1070 cm^−1^ and corresponds to the O-P-O symmetric stretching mode [28,29,30,32,33,34]. The weak band at 1092 cm^−1^ band is due to the antisymmetric O-P-O stretching vibration [30,33,34]. To summarize, most of the spectral shifts observed upon tyrosine phosphorylation are very similar to those observed in previously reported Raman studies [27,31,35,36]. While the different types of vibrations can be slightly different in position and shape in the proteins due to their sensitivity to the microenvironment, their band positions vary by up to only to few cm^−1^ in the Raman spectra of the proteins compared to the reference tyrosine and phosphorylated tyrosine.

Having obtained the reference Raman fingerprint of tyrosine phosphorylation, we focused on the Raman spectral changes arising in the proteins due to tyrosine phosphorylation in normal and cancerous breast, small intestine and brain tissues.

**Figure 3:**
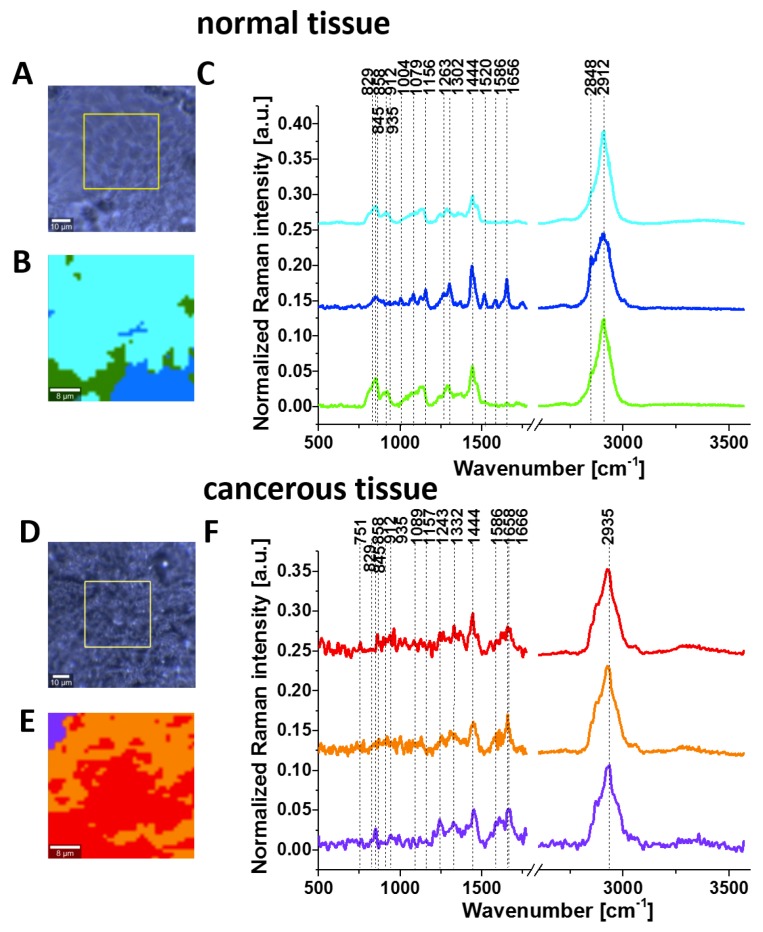
Typical images of normal breast tissue: microscopy image (**A**); Raman image (40 μm x 40 μm) obtained by cluster analysis (**B**) and the vibrational Raman spectra in the frequency region of 500–3600 cm^−1^ (**C**). Images of cancerous breast tissue (invasive ductal carcinoma G3 P134): microscopy image (**D**); Raman image (40 μm x 40 μm) obtained by cluster analysis (**E**) and the vibrational Raman spectra in the frequency region of 500–3600 cm^−1^ (**F**). The line colours of the Raman spectra correspond to the colours of the Raman maps, thickness of samples: 16 μm.

Figure 3 shows the Raman images and Raman spectra for the normal and cancerous breast tissue for invasive ductal carcinoma (G3), where the abnormal cells infiltrate the extracellular matrix (ECM).

The tumour microenvironment has a very important influence in cancer development. This aspect of the dynamic nature of breast cancer and excessive fibrosis have been discussed by Abramczyk’s group in several papers. Abramczyk and colleagues showed that Raman biochemical mapping of human cancer tissues reveals unique Raman fingerprints that discriminate normal and cancer cells [37], cancer phenotype [38], lipid reprograming [39], epigenetic modifications [19,40], angiogenesis in cancer induced by increased collagen crosslinking in breast cancer [41] and glycome profile [42]. In this paper, we mainly concentrate on the fingerprint region of Raman spectra (500–1800 cm^−1^) because the vibration of phosphorylated proteins occurs in this region.

**Figure 4:**
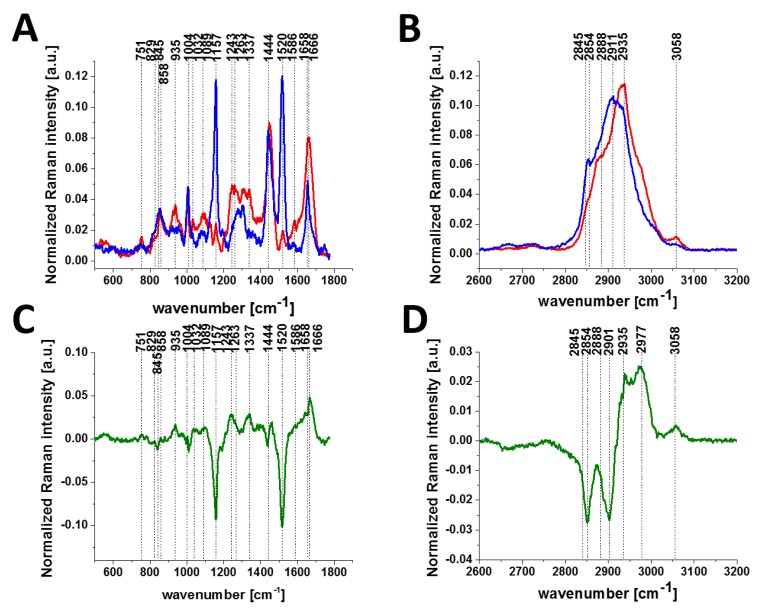
Average Raman spectra for cancerous (red line) and normal breast (blue line) tissues in the fingerprint region (**A**) and in the high-frequency region (**B**) and difference spectra (cancerous-normal tissue) in the fingerprint region (**C**) and in the high-frequency region (**D**). Invasive ductal carcinoma G3, P134.

Figure 4 shows the average Raman spectra for cancerous (invasive ductal carcinoma G3) and normal breast tissues in the fingerprint region (Figure 4A) and in the high-frequency region (Figure 4B), and difference spectra (cancerous-normal tissue) in the fingerprint region (Figure 4C) and in the high-frequency region (Figure 4D), respectively.

It is evident from Figure 3 and Figure 4 that invasive cancer development produces many spectral changes in the Raman spectrum compared to the normal breast tissue. The Raman spectra in Figure 4 provide a multitude of information about the lipid phenotype [20,37,38] and epigenetic modifications [19].

The most significant differences between the normal and cancerous breast tissues are related to the vibrations of carotenoids (1157, 1520 cm^−1^), DNA (751 cm^−1^), lipids (2845, 2888 cm^−1^), proteins (1666, 2935 cm^−1^) and phosphorylated proteins. The results for carotenoids, lipids and DNA presented here support our earlier results that provided clear evidence for the overexpression of carotenoids and lipids and the lower expression of DNA in normal breast tissue. Details can be found in recent publications from our group [19,20,38,40].

Here, we will focus only on the phosphorylation effects. They clearly reveal themselves in Figure 4. First, the invasive ductal carcinoma exhibits a Raman peak at 1586 cm^−1^ corresponding to the phosphorylated protein. The peak at 1586 cm^−1^ is significantly less intense in the normal tissue compared to the cancerous one. Second, the characteristic doublet (829 cm^−1^ and 845 cm^−1^ of tyrosine is clearly visible in Figure 4A and corresponds to a Fermi resonance between the first overtone of the aromatic out-of-plane ring bend and the aromatic ring breathing fundamental [28]). The band associated with the O-P-O stretching mode of the phosphate group at 858 cm^−1^ from phosphorylated tyrosine (partially overlapping with phosphate group from DNA) is revealed in the cancerous tissue. Third, vibrations of the PO_4_^3-^ phosphate group of the phospholipids are observed at 1089 cm^−1^ and correspond to the O-P-O symmetric stretching mode [28]. Fourth, the band at 1263 cm^−1^ corresponding to Amide III is shifted to 1243 cm^−1^ upon tyrosine phosphorylation, possibly indicating a secondary structure change from the alpha helix (or random coil) to a beta-sheet-like structure [27,28] in the cancerous tissue. Fifth, a band at 935 cm^−1^ corresponding to glycogen is clearly observed in the cancerous tissue in contrast to the normal tissue. Sixth, the shift in the PO_4_^−^ phosphate group peaks from 1070 cm^−1^ in the phosphorylated tyrosine (Figure 2) to 1089 cm^−1^ in the breast tissue (Figure 4A), and reflects the dominant contribution from the phospholipids of the membranes.

In view of the results presented in Figure 4, it can be stated that the characteristic Raman vibrations shown in Figure 4 provide evidence that the phosphorylation of tyrosine residues introduces appreciable spectral changes in the Raman spectrum of the invasive ductal carcinoma compared to the normal tissue. Therefore, the Raman spectroscopy has the potential to be used as a diagnostic assay for phosphorylation in living cells and tissues.

To check if the phosphorylation effects are also observed by Raman spectroscopy for other types of cancer, we compared normal and cancerous human small intestine tissues.

**Figure 5:**
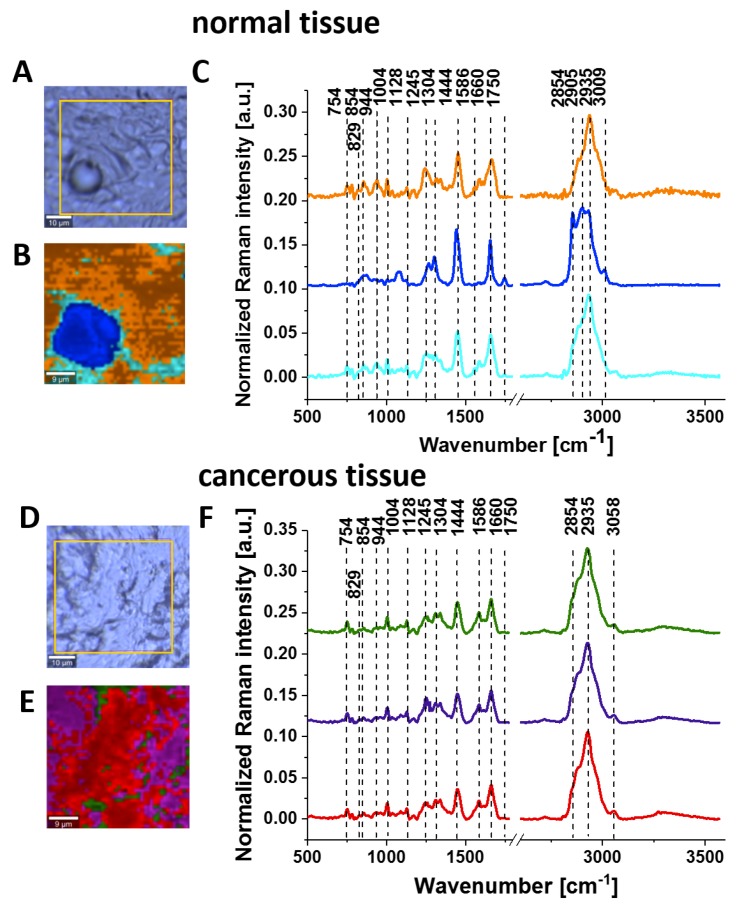
Typical images of normal small intestine tissue (PZK): microscopy image (**A**); Raman image (45 μm × 45 μm) obtained by cluster analysis for the low-frequency region (**B**) and the vibrational Raman spectra in the wide frequency range of 500–3600 cm^−1^ (**C**). Images of cancerous small intestine tissue (Adenocarcinoma G2, pT3, small intestine passing in the large intestine tumour infiltration at ileocecal valve PZK): microscopy image (**D**); Raman image (45 μm × 45 μm) obtained by cluster analysis for the low-frequency region (**E**) and the vibrational Raman spectra in the wide frequency range of 500–3600 cm^−1^ (**F**). The line colours of the Raman spectra correspond to the colours of the Raman maps, thickness of samples: 16 μm.

Figure 5 shows the typical Raman images and Raman spectra of normal and cancerous human small intestine tissues.

**Figure 6:**
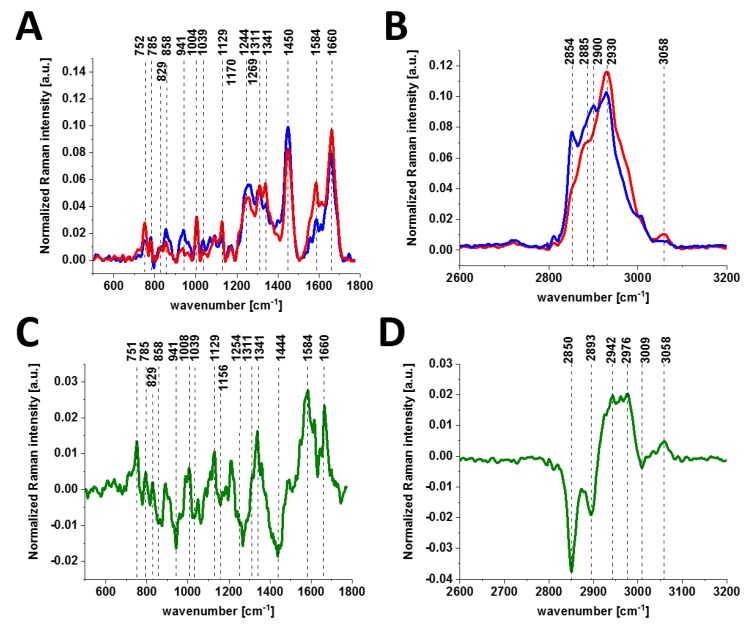
Average Raman spectra of the small intestine in normal tissue (blue line) and cancerous tissue (red line) in the fingerprint region (**A**) and in the high-frequency region (**B**); difference spectra (cancerous-normal tissue) in the fingerprint region (**C**) and in the high-frequency region (**D**).

Figure 6 shows the comparison of the average Raman spectra of the normal and cancerous human small intestine tissues.

As observed from the comparison in the Raman fingerprint region, the enhanced tyrosine phosphorylation profile is clearly visible for the small intestine cancer. First, the normalized Raman intensity of the band at 1586 cm^−1^ is higher for the cancer tissue than for the normal tissue, indicating a higher concentration of phosphorylated tyrosine in the epithelial cells of the cancerous small intestine. Second, the band of the nonphosphorylated tyrosine at 858 cm^−1^ has a much higher Raman intensity in the normal small intestine tissue than in the cancerous tissue. In contrast, the intensity of the band at 1129 cm^−1^ corresponding to glucose [28] is much stronger in the small intestine cancer tissue than in the normal tissue, illustrating a higher uptake of glucose in cancer. The band at 1170 cm^−1^ corresponds to the C-H in-plane bending mode of tyrosine and phosphorylated tyrosine [28].

To check the tyrosine phosphorylation in metabolic regulation in brain tumours, we studied the Raman vibrations of phosphorylated proteins in brain tissues.

**Figure 7:**
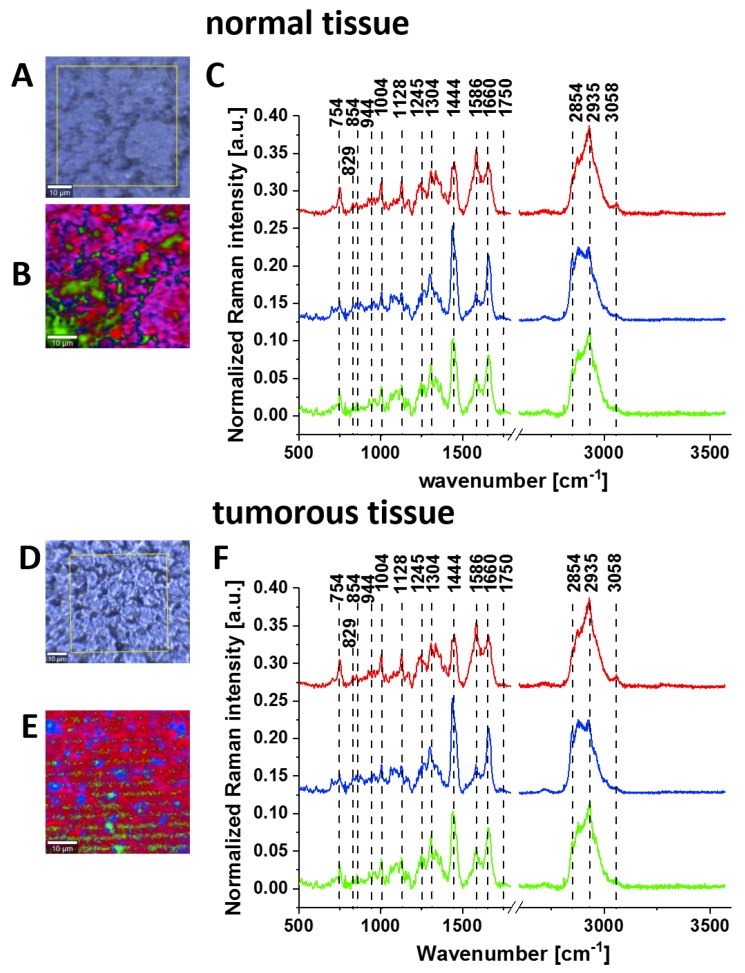
Typical images of normal brain tissue: microscopy image (**A**); Raman image (50 μm x 50 μm) obtained by cluster analysis for the low-frequency region (**B**) and the vibrational Raman spectra in the wide frequency range of 500–3600 cm^−1^ (**C**). Images of tumorous brain tissue (medulloblastoma WHO IV): microscopy image (**D**), Raman image (50 μm × 50 μm) obtained by cluster analysis for the low-frequency region (**E**) and the vibrational Raman spectra in the wide frequency range of 500–3600 cm^−1^ (**F**). The line colours of the Raman spectra correspond to the colours of the Raman maps, thickness of samples: 16 μm.

Figure 7 shows the typical Raman images and the vibrational Raman spectra in the wide frequency range of 500–3600 cm^−1^ of the normal and the tumorous (medulloblastoma WHO (World Human Organization) IV) human brain tissues.

**Figure 8:**
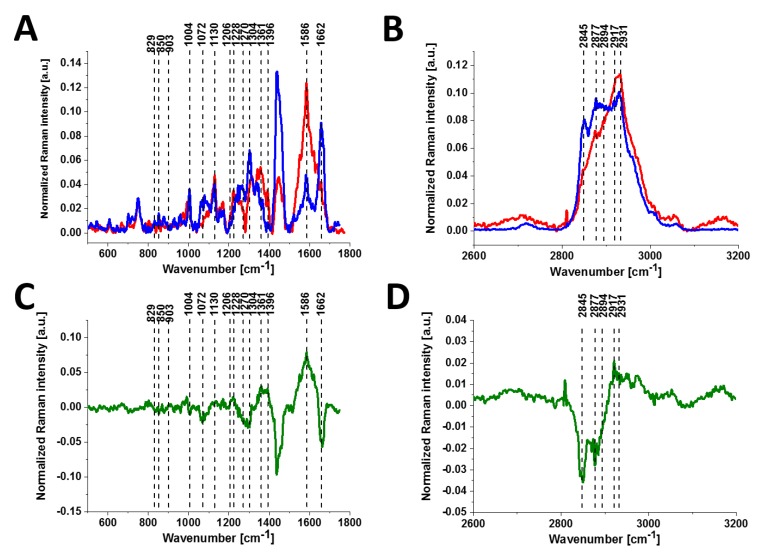
Average Raman spectrum for tumorous (human, medulloblastoma WHO IV) tissue from the brain (red line) and normal human brain (blue line) in the fingerprint region (**A**) and in the high-frequency region (**B**); difference spectra (cancerous-normal tissue) in the fingerprint region (**C**) and in the high-frequency region (**D**).

Figure 8 shows the average Raman spectra for a normal human brain and human medulloblastoma (grade IV). Many significant biochemical differences between the normal and tumour structures can be observed in Figure 8. It is evident from Figure 8 that the brain tissue has the same universal characteristic Raman features of tyrosine phosphorylation like breast and small intestine tissues. First, we observe the band at 1270 cm^−1^ corresponding to Amide III of the normal tissue, which is shifted to 1228 cm^−1^ upon the phosphorylation in the cancer tissue. The most spectacular difference is observed at 1586 cm^−1^ with a very strong band of phosphorylated protein in the malignant tissue. This band is much less intense in the normal tissue, indicating concentration of phosphorylated tyrosine is lower in the normal brain than in the cancer tissue. The Raman band at approximately 1586 cm^−1^ was observed in various biological systems and was assigned to amide II (CH_2_ deformation in glioblastoma [43], nucleotides [44,45] pyrimidine ring of nucleic acids [46] phenylalanine (C=C bending) [47,48,49,50,51] lipids [52] and phosphorylated tyrosine [27,31,35,36]. Our results for tyrosine and phosphorylated tyrosine presented in Figure 2 clearly show that the Raman band at 1586 cm^−1^ corresponds to phosphorylated tyrosine. Tyrosine phosphorylation activity evident from Figure 8 for medulloblastoma, which is a paediatric malignant primary brain tumour, supports the recent reports on the role of tyrosine phosphorylation inhibitors in therapy for medulloblastoma [53].

So far, we have analysed global tyrosine phosphorylation in the cells from the epithelial breast, small intestine digestive tract, and brain tissues. Now, we will focus on a single glial cell of glioblastoma (U-87 MG) that has an epithelial-like morphology. We employed Raman techniques to determine the location and biochemical composition of the cell organelles, particularly lipid droplets, nucleus, membrane and subcellular localization of the phosphorylated proteins.

**Figure 9:**
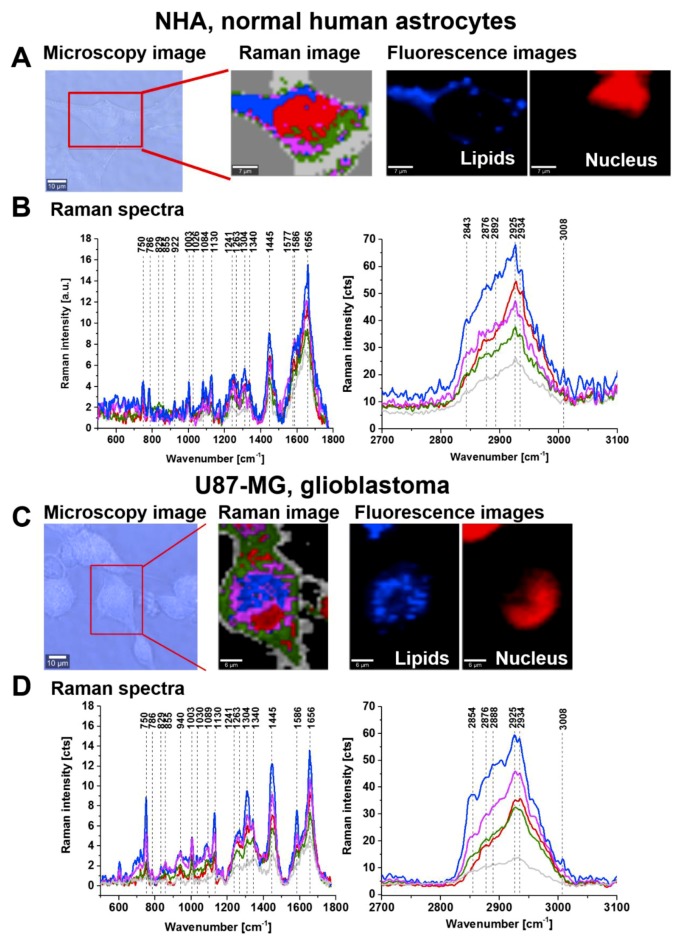
Live cells of human normal astrocytes (NHA) and glioblastoma (U-87 MG) cells imaged by microscopy, Raman imaging (NHA: 45 × 30 μm, U87MG: 30 × 45 μm) and fluorescence staining of lipids (Oil Red O) and nucleus (Hoechst 33342) (**A**,**C**). Raman spectra of lipid droplets (blue), nucleus (red), cell membrane (light grey), cytoplasm (green) and mitochondria (magenta) in the spectral ranges of 500–1800 cm^−1^ and 2700–3100 cm^−1^ (**B**,**D**). The laser excitation power was 10 mW, and the collection time was 0.5 for the Raman image (laser excitation 532 nm) and 0.1 s for the fluorescence images (laser excitation 532 nm for Oil Red O fluorescence and 355 nm for Hoechst 33342 fluorescence). Raman/fluorescence images were recorded with a spatial resolution of 1 μm.

Figure 9 shows the typical live cells of normal human astrocytes (NHA) and glioblastoma (U-87 MG) imaged by microscopy, Raman imaging and fluorescence imaging after staining.

The Raman image presented in Figure 9 shows the localization of lipid droplets (blue), nucleus (red), cell membrane (light grey), cytoplasm (green) and mitochondria (magenta). Figure 9B,D shows the Raman spectra from the areas inside the cell localized in/on lipid droplets, nucleus, mitochondria and membrane of the cell. The analysis of Raman results show that characteristic bands at approximately 1586 cm^−1^ and 1258 cm^−1^ assigned to phosphorylated tyrosine can be observed, and these are particularly intense in lipid droplets and mitochondria. The nucleus shows many characteristic nucleic acid (guanine, adenine) peaks at 1577 cm^−1^ and 1241 cm^−1^ corresponding to the pyrimidine ring in nucleic acids [28] and at 1241 cm^−1^ corresponding to the DNA pyrimidine bases (cytosine, thymine). The peak at 1089 cm^−1^ corresponds to the O-P-O stretching mode in the nucleic acids of the nucleus. The peak at 786 cm^−1^ observed in the nucleus corresponds to DNA (cytosine, uracil, thymine). The nucleus-associated phosphorylated tyrosine proteins are less visible than the proteins localized on lipid droplets and in the mitochondria of the cell.

## 3. Discussion

To access the diagnostic potential of Raman spectroscopy in the monitoring of tyrosine phosphorylation in cancer cells from the vibrational signatures, we calculated the Raman intensity ratios for the characteristic vibrations related to phosphorylated and non-phosphorylated proteins. As the Raman intensity is proportional to the concentration, the ratios in Table 1 measure the concentration ratios of phosphorylated to non-phosphorylated proteins and the ratio of phosphorylated to global proteins concentration in normal and cancerous human breast, small intestine and brain tissues.

To evaluate the significance of statistical evidence in discrimination between the normal and cancerous/tumorous tissues, the results in Table 1 and Table 2 are based on a set of 35 clinical unprocessed fresh human breast, small intestine and brain samples (the number of normal samples was equal to the number of cancerous/tumorous samples).

Table 2 shows the sensitivity and specificity for each studied type of cancer obtained by Raman-based assay of protein phosphorylation. As presented in Table 2, sensitivity and specificity were obtained from a partial least squares discriminant analysis (PLSDA).

**Figure 10:**
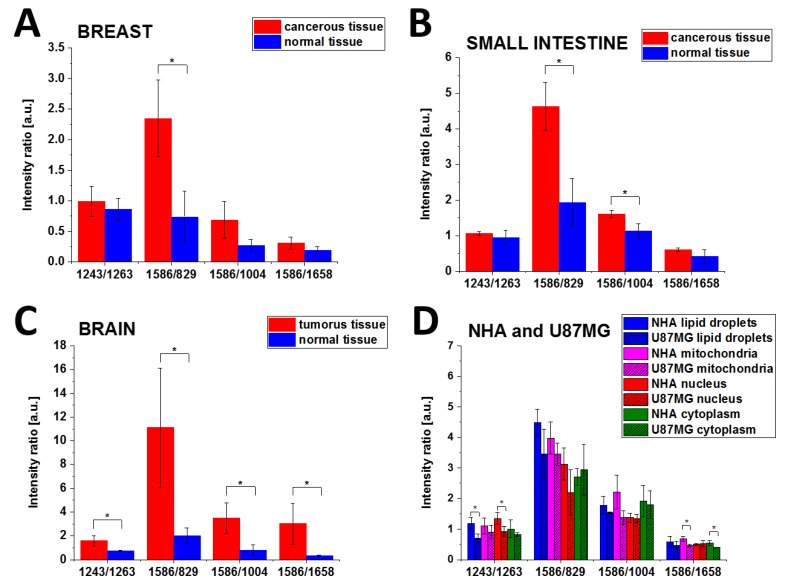
Schematic representation of the Raman intensity ratios, I_1243_/I_1263_, I_1586_/I_829_, I_1586_/I_1004_ and I_1586_/I_1658_, for the analysed breast (**A**), small intestine (**B**), brain samples (**C**), NHA and U87-MG cell lines (**D**). The one-way ANOVA using the Tukey test was used to calculate the value significance, star * denotes that the differences are statistically significant, *p* Values ≤ 0.05 were accepted as statistically significant.

Figure 10 presents the schematic representation of the I_1243_/I_1263_, I_1586_/I_829_, I_1586_/I_1004_, and I_1586_/I_1658_ Raman intensity ratios for normal and cancerous breast (invasive ductal carcinoma G3) and small intestine (adenocarcinoma G2), normal and tumorous brain (medulloblastoma) tissue samples and NHA, U87-MG cell lines.

It is evident from Table 1 and Figure 10A–C that the Raman intensity ratio: I_1243_/I_1263_, I_1586_/I_829_, I_1586_/I_1004_ of the phosphorylated proteins to nonphosphorylated proteins is much higher for the cancerous tissue than in the normal tissue for all studied organs. Using the term of overexpression that predominates in biological studies to describe a process of making too many copies of a protein or other substance, we can claim that the overexpression of tyrosine phosphorylation monitored by Raman vibrations at 1586 and 829 cm^−1^ is a universal characteristic signature of metabolic regulation in breast and small intestine cancers and brain tumours, and indicates that overexpression of phosphorylated proteins play an important role in cancer development.

The same analysis was performed for Raman spectra of different organelles in NHA and U-87 MG cells. Table 3 shows the I_1243_/I_1263_, I_1586_/I_829_, I_1586_/I_1004_ and I_1586_/I_1658_ Raman intensity ratios for the normal human astrocytes (NHA) and glioblastoma U-87 MG cells.

Figure 10 shows that the best Raman biomarker is the 1586/829 ratio for breast and small intestine cancers. For that of the brain tumour tissue, all ratios: I_1243_/I_1263_, I_1586_/I_829_, I_1586_/I_1004_ and I_1586_/I_1658_ discriminate normal and cancerous human tissues showing the statistical significance at *p* Values ≤ 0.05.

One can see from Table 3 that the concentrations of phosphorylated proteins compared to nonphosphorylated proteins are similar for lipid droplets and mitochondria. The nucleus phosphorylated tyrosine proteins are less visible than the proteins localized on lipid droplets and in the mitochondria of the glioblastoma cell (I_1586/829_ lipid droplets; 3.46 ± 0.81, I_1586/829_ mitochondria 3.45 ± 0.37, I_1586/829_ nucleus 2.20 ± 0.75).

The unique capability of Raman imaging to perform chemical imaging with high spatial resolution without the addition of any exogenous tags has paved the way for quantitative investigations of lipid reprogramming in cancer cells and of epigenetic modifications with spatial details inaccessible for other methods [38,40]. Recently we have shown that Raman spectroscopy enables new and deeper investigations of lipid phenotype, fatty acid composition, and spatial distribution of lipids and LDs inside tumours [37,38]. Here, we focused on the proteins in the cytoplasm and the proteins coating the surface of lipid droplets, mitochondria, nuclei and membranes of glioblastoma cells. Figure 9 shows that we can monitor the activities of both nonreceptor tyrosine kinase enzymes that are located in the cytoplasm, and the receptor kinases that are associated with a cell-surface receptor containing a transmembrane domain.

The localization of receptors that show a tyrosine kinase activity on lipid droplets reveals the unexpected role of lipid droplets in cancer cells. The results obtained in this work show that lipid droplets are functional organelles that are not only responsible for fat storage, but are also involved in lipid metabolism with enhanced tyrosine kinase activity and participate in cancer development. We are only now beginning to gain insight into their role, but our results indicate the important role of lipid droplets in cancer development.

Our results support the new discovered functions of the lipid droplets that include activity in the synthesis and storage of inflammatory mediators in cancer cells [37,54]. The proteins associated with lipid droplets cover the surface of the membrane monolayer and participate in the lipid droplets formation, growth, transport, and catabolism [54]. It has been found that some protein components that are present on the lipid droplet surfaces are derived from endoplasmic reticulum (ER) and the enzymes involved in TAG and CE synthesis reside on the ER membrane [55,56]. The LD-associated proteins that we observe in Figure 9 can be either cytosol-derived proteins or ER-derived proteins [57]. A strong link between lipid droplets, ER and mitochondria has been reported [58] because lipid droplets are often found in close contact with both ER cisternae and mitochondria where the fatty acids oxidation (FAO) occurs.

The role of lipid droplets in mechanisms of cancer development is only beginning to be explored. The localization of the phosphorylated tyrosine proteins on lipid droplets shown in Figure 9A reveals their important role in human cancers. Recently, we showed that higher number of lipid droplets is associated with more aggressive tumours [37]. It was reported that tumour aggressiveness may be related to the accumulation of proteins involved in tumourigenesis, such as phosphatidylinositol-4,5-bisphosphate 3-kinase (PI3K), extracellular signal-regulated kinase 1 and 2 (ERK1 and ERK2), and caveolins in the lipid droplets of different cancer cells [59,60,61].

In sum, it can be concluded that our discoveries help to promote Raman-based diagnostic value in cancer research. The results presented in the paper provided evidence that for all types of cancers and tumours studied in this paper (breast, small intestine and brain), enhanced phosphorylated tyrosine production occurs. It suggests that metabolic effects of enhanced tyrosine phosphorylation in cancer cells can be treated as the universal hallmarks of cancerogenesis.

Our results support the previous findings on the role of phosphorylation studied by the methods of conventional biology [62,63,64].

**Figure 11:**
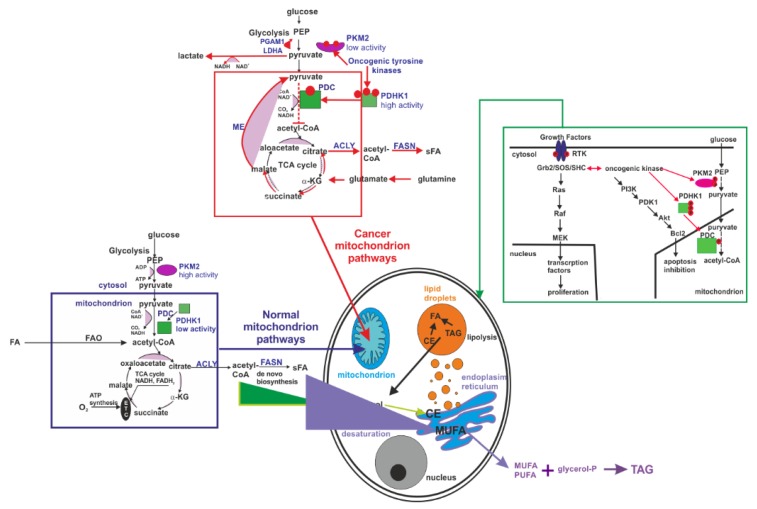
Effect of tyrosine phosphorylation of PDHK1 and PKM2 on the fates of pyruvate and acetyl-CoA in normal and cancer cells (modified according to [5]). (Blue frame) In normal cells, acetyl-CoA is formed in by glycolytic pathway and fatty acid oxidation (FAO). Phosphoenolpyruvate (PEP) generated during glycolysis is converted into pyruvate by the highly active tetrameric isoform M2 of pyruvate kinase (PKM2). Pyruvate enters the mitochondria and is transformed into acetyl-CoA by dehydrogenase complex (PDC) as a result of irreversible oxidative decarboxylation. Then, acetyl-CoA is primarily oxidized to produce CO_2_ and generate NADH and FADH2 in the tricarboxylic acid (TCA) cycle. Reduced nucleotides are the fuel of the mitochondrial electron transport chain (ETC) that is used to create cellular energy in the form of ATP. A small fraction of acetyl-CoA is used to synthesize FA and cholesterol. (Red frame) Oncogenic tyrosine kinases (BCR-ABL, JAK2) highly expressed in cancer cells perform tyrosine phosphorylation of pyruvate dehydrogenase kinase 1 (PDHK1). PDHK1 phosphorylates pyruvate dehydrogenase alpha 1 (PDHA1), which is a subunit of the PDC. The result of PDHA1 phosphorylation is the inhibition of the conversion of pyruvate to acetyl-CoA and an increase in lactate production. Additionally, oncogenic tyrosine kinases phosphorylate PKM2, which causes a reduction of its activity as a result of the conversion of the tetrameric form to the dimeric form. The result of these tyrosine phosphorylation events is the suppression of mitochondrial pyruvate metabolism. Therefore, cancer cells promote the utilization of TCA intermediates derived from glutamine metabolism (α-ketoglutarate, α-KG) to provide citrate for fatty acids biosynthesis. Additional source of acetyl-CoA is the enhanced FAO. (Green frame) Activation of a growth factor receptor involves its dimerization and autophosphorylation of key tyrosine residues and leads to the activation of downstream signaling cascades, including the Ras/Raf/MEK pathway, which promotes cell proliferation. Oncogenic tyrosine kinases overexpressed in cancer cells also stimulate PI3K/Akt pathway, resulting in inhibition of apoptosis. Oncogenic tyrosine kinases, also phosphorylate enzymes (PKM2, PDHK1), engaged in pyruvate and acetyl-CoA metabolism. Abbreviations: ATP-citrate lyase (ACLY), B-cell lymphoma 2 (Bcl2), cholesteryl ester (CE), fatty acid (FA), fatty acid synthase (FASN), growth factor receptor-bound protein 2 (Grb2), lactate dehydrogenase (LDHA), malic enzyme (ME), mitogen activated protein kinase (MEK), monounsaturated fatty acid (MUFA), phosphatidylinositol 3-kinase (PI3K), phosphoglycerate mutase (PGAM1), 3-Phosphoinositide-dependent kinase 1 (PDK1), polyunsaturated fatty acid (PUFA), protein kinase B (Akt), receptor tyrosine kinase (RTK), Src homology 2 domain-containing (SHC), son of sevenless (SOS).

To obtain an in-depth mechanistic connection to the molecular basis of cancer, we will combine our findings with current interpretations on the mechanisms of phosphorylation in the literature. Numerous studies have been performed to determine the differences in the mechanisms of phosphorylation occurring under normal physiological conditions and enhanced tyrosine kinase activity in the dysregulation of cell growth, aberrant cell signalling, and altered metabolisms in tumour cells [5,65,66,67], as illustrated in Figure 11.

Although the interplay between glucose metabolism in cancer, enhanced de novo lipid synthesis and amplified tyrosine kinase phosphorylation activity remains poorly understood, the following mechanism presented in Figure 11 can explain the Raman signatures of the amplified tyrosine phosphorylation activity in cancer reported in this paper.

It is commonly accepted that cancer cells display significant metabolic modifications compared to normal cells that are related to:

(1) An increased uptake of glucose with mechanisms of ATP synthesis through glycolysis (Warburg effect) [68].

(2) Enhanced de novo lipid synthesis, where fatty acid synthase (FASN) and sterol regulatory element-binding protein (SREBP) family are regarded as the most essential functional factors in many human cancers [69,70,71,72,73,74,75].

(3) Amplified tyrosine kinase phosphorylation activity, with key regulatory components in signal transduction pathways (such as Ras-Raf-MAPK or PI3-K/AKT pathways) that are important in the control of cell growth, proliferation, differentiation, and transformation [23,76].

Briefly, in the first step, some receptors (e.g., EGFR) trigger the signalling pathway that leads to the enhanced activity of tyrosine kinase and tyrosine phosphorylation of PDHK1 in mitochondria (partially in the nucleus) and PKM2 in cytosol of cancer cells that results in the modification of pyruvate and acetyl-CoA fates compared to the normal cells.

In normal cells during the glycolysis, phosphoenolpyruvate (PEP) is formed and then is converted into pyruvate by highly active tetrameric isoform M2 of pyruvate kinase (PKM2). Pyruvate enters the mitochondria and is transformed into acetyl-CoA by dehydrogenase complex (PDC) as a result of irreversible oxidative decarboxylation. Next, acetyl-CoA is oxidized to produce CO2 and generate NADH and FADH2 in the tricarboxylic acid (TCA) cycle. Reduced nucleotides are the fuel of the mitochondrial electron transport chain (ETC) used to create cellular energy in the form of ATP [5].

In cancer cells, oncogenic tyrosine kinases (BCR-ABL, JAK2) are highly expressed and perform tyrosine phosphorylation of pyruvate dehydrogenase kinase 1 (PDHK1). PDHK1 phosphorylates pyruvate dehydrogenase alpha 1 (PDHA1) that is a subunit of the PDC. PDHA1 phosphorylation results in the inhibition of the conversion of pyruvate to acetyl-CoA and in the increase in lactate production. Additionally, oncogenic tyrosine kinases phosphorylate PKM2, causing the lowering of its activity as a result of the conversion of the tetrameric form to a dimeric form. Blocking of mitochondrial pyruvate metabolism may promote the utilization of the TCA intermediates derived from glutamine metabolism (α-ketoglutarate, α-KG) to provide citrate for fatty acids (FA) biosynthesis [5]. The enhanced de novo lipid synthesis balances energy deficit due to replacing oxidative phosphorylation by lactate fermentation and decreased availability of ATP in cancer cells. The enhanced de novo lipid synthesis is clearly supported by our previously reported results [39,40] and is also evident from the results obtained in the present work.

Although this model explains the enhanced de novo lipid synthesis, it is still unclear how the Warburg effect with enhanced glycolysis in the cytosol is related to the suppressed mitochondrial pyruvate metabolism. It was found that PDHK1 can also be phosphorylated by other cytosolic proteins involved in glycolysis, namely PGK1 [77]. It was demonstrated that EGFR triggers the signalling pathway in which phosphorylation of serine 203 in PGK1 occurs and PGK1 translocate from the cytosol to the mitochondria where it phosphorylates PDHK1. This phosphorylation can result in the inhibition of the mitochondrial pyruvate metabolism and amplifies lactate production, thereby promoting tumour development [77].

## 4. Materials and Methods

### 4.1. Chemicals

Tyrosine (T3754), phosphotyrosine (P9405), Hoechst 33342 (B2261) and Oil Red O (O0625) were purchased from Sigma Aldrich (St. Louis, MO, USA). Mitotracker Red CMXRos (M7512) was purchased from Thermo Fisher Scientific (Waltham, MA, USA). We have prepared 10^−2^ M solutions of tyrosine and phosphotyrosine in water. Next, a 20-μL drop of each solution was deposited on CaF_2_ windows and air-dried.

### 4.2. Patients

Raman spectra and images were obtained from fresh human tissue sections, namely 14 cancerous breast tissue samples, 11 brain tumour samples and 10 small intestine cancerous samples. The total number of the control samples was the same as the number of the cancerous samples. The control samples were taken from the negative margin of the tissue removed during the surgery. All of the procedures for human breast and small intestine tissues were conducted under a protocol approved by the institutional Bioethical Committee at the Medical University of Lodz (RNN/323/17/KE/17/10/2017). Written consent was obtained from the participating patients. All of the brain experiments were performed in compliance with the relevant laws and guidelines of the Bioethical Committee at the Polish Mother’s Memorial Hospital Research Institute in Lodz (53/216) and of the Ministry of Health of the Republic of Poland.

The ex vivo samples were obtained during the resection surgery from the tumour mass and the safety margin. All of the tissue specimens were frozen and stored at −80°C. Prior to the measurements, the frozen samples were cryosectioned at −20°C with a microtome (Microm HM 550, Sermed, Thermo Scientific, Waltham, MA, United States) into a few 16-µm-thick slices for Raman measurements and were placed onto calcium fluoride windows (CaF_2_, 25 × 1 mm).

### 4.3. Cell Line

U87-MG (brain glioblastoma, morphology: epithelial, HTB-14) and NHA (Clonetics normal human astrocytes, CC-2565) cell lines were purchased from ATCC (American Type Culture Collection, LGC Standards, Teddington

Middlesex, UK.) and Lonza (Lonza Walkersville, Inc, Walkersville, MD 21793, USA), respectively. The cells were cultured in a monolayer culture using the techniques described in the ATCC and Lonza protocols. Raman spectra of living cells were recorded. The procedure for the Raman measurements of the cells was as follows: U-87 MG or NHA cells were seeded onto CaF_2_ windows (25 × 1 mm). After 24 h of incubation on the CaF_2_, the cells were rinsed with a phosphate-buffered saline (PBS, SIGMA P-5368, pH 7.4 at 25°C, *c* = 0.01 M) to remove any residual medium. The Raman confocal measurements were performed immediately after the preparation of the samples without staining. For identification of the organelles, the cells were stained with Oil Red O, Hoechst 33342 and Mitotracker Red CMXRos according to the manufacturer protocols for the detection of cytoplasmic lipid droplets, nucleus, and mitochondria, respectively. The confocal fluorescence measurements were performed for comparison with the Raman images.

### 4.4. Equipment

Raman spectra and images were recorded using a confocal Raman microscope (WITec (alpha 300 RSA+), Ulm, Germany) in the Laboratory of Laser Molecular Spectroscopy, Lodz University of Technology, Poland. The Raman microscope consisted of an Olympus microscope (Olympus Düsseldorf, Germany) a UHTS (Ultra-High-Throughput Screening) monochromator (WITec, Ulm, Germany) and a thermoelectrically cooled CCD camera ANDOR Newton DU970N-UVB-353 (EMCCD (Electron Multiplying Charge Coupled Device, Andor Technology, Belfast, Northern Ireland) chip with 1600 × 200 pixel format, 16 μm dimension each) at −60°C with full vertical binning. A 40× water immersion objective (Zeiss, W Plan-Apochromat 40×/1.0 DIC M27 (FWD = 2.5 mm), VIS-IR) was used for cell lines measurements and a 40× objective (NIKON CFI Plan Fluor C ELWD (Extra-Long Working Distance) 40×: N.A. 0.60, W.D. 3.6–2.8 mm; DIC-M, C.C.0-2) was used for the Raman measurements of chemicals and tissues. The excitation laser at 532 nm (Raman measurements, Oil Red O fluorescence, Mitotracker Red CMXRos fluorescence) or 355 nm (Hoechst 33342 fluorescence) was focused on the sample to the laser spot of 1 μm and was coupled to the microscope via an optical fibre with a diameter of 50 μm. The average laser excitation power was 10 mW, and the collection time was 0.5 and 1 s for Raman images and 2 s and 10 accumulations for the single Raman spectra in the spectral ranges of 200–1800 cm^−1^ and 2100–3600 cm^−1^. Raman images were recorded with a spatial resolution of 1 × 1 μm. The Raman microspectrometer was calibrated every day prior to the measurements using a silica plate with a maximum peak at 520.7 cm^−1^.

For Hoechst 33342 fluorescence images, a 355 nm diode laser (WITecec, Ulm, Germany) with an excitation power of 5 mW and acquisition time of 0.1 s with the spectral centre at 7200 cm^−1^ was used. For Oil Red O and Mitotracker Red CMXRos fluorescence images, a 532 nm diode laser with an excitation power of 10 mW and collection time of 0.1 s was used with the spectral centres at 3500 cm^−1^ and 2100 cm^−1^, respectively.

### 4.5. Data Processing

The raw Raman spectra and Raman images were analysed in the ranges of 500–1800 cm^−1^ and 2600–3200 cm^−1^. Raman data were preprocessed using the WITec Control/Project Four 4.1 package. The sharp spikes attributed to cosmic rays were removed by using frequency and spatial filtering. The corrected Raman spectra were smoothed by using a Savitzky and Golay procedure. Background subtraction, and the elimination of the Rayleigh scattering and the Raman scattering of the CaF_2_ support were performed. The spectra from a statistically significant number of samples were collected to calculate the Raman average spectra and to perform statistical analyses. Average Raman spectra were calculated as an arithmetic average of the recorded Raman spectra.

The Raman images were created by the K-means Cluster Analysis (CA) [38]. For comparisons, Raman spectra were vector normalized in the Origin software (divided by the norm). The N-dimensional Raman normalized vector $\hat{X}$ is given by $\hat{X}=\frac{X}{\left|X\right|}$ where |*X*| is the norm of *X*. The normalization allows us to compare the Raman spectra from different measurements and samples.

### 4.6. Statistics

The number of times the experiment was repeated is equal to the number of Raman spectra taken for statistics to calculate the average and SD. The typical number for each cancer type was minimum 1600 Raman spectra per image times the number of patients that gives around 16,000 Raman spectra for statistics. The data was expressed as a mean value ± standard deviation (SD). All data were analyzed using Origin software with the implemented one-way ANOVA using the Tukey test to calculate the value significance. *p*-Values ≤ 0.05 were accepted as statistically significant. A partial least squares discriminant analysis (PLSDA) with receiver operating characteristic area under the curve (ROC AUC) was carried out with MATLAB and the PLS_Toolbox (Eigenvector Research). The PLSDA was applied in a cross-validation scheme of venetian blinds with three splits.

## 5. Conclusions

We developed, for the first time, a label-free Raman method for detecting the spectral changes that arise in proteins due to phosphorylation in human breast, small intestine cancers, and brain tumourous tissues and in single glioblastoma cells.

Our results based on credible statistics (around 16,000 spectra for each type of cancer) proved that Raman spectroscopy and imaging have a potential to be used as screening functional assays to detect phosphorylated target proteins and will help researchers to understand the role phosphorylation plays in cellular processes and cancer progress.

We showed that Raman spectroscopic technique provides real time biochemical profile of tissues or organelles in single cells without disrupting cells to break open and release the cellular structures.

We showed that Raman-based method is an effective tool for monitoring phosphorylation alterations in the human tissue compared to the normal tissue (control) by analyzing the vibrations of functional groups involved in the metabolic processes occurring in the tissue.

We showed that the band at 1586 cm^−1^, corresponding to phosphorylated tyrosine, plays a pivotal role as a Raman biomarker of the phosphorylation status in aggressive cancers. We found that the best Raman biomarker of phosphorylation is the 1586/829 ratio showing the statistical significance at *p* Values ≤ 0.05.

We found overexpression of phosphorylated tyrosine identified by Raman spectroscopy in a variety of human cancers studied in this paper, namely, ductal cancer (grade III), small intestine (adenocarcinoma, grade II), brain tumour of medulloblastoma (WHO grade IV) and glioblastoma. 

The aberrant phosphorylated tyrosine in human tumours cell line has been found to be localized in mitochondria and lipid droplets. The results of this study proved that the localization of phosphorylated proteins can be monitored and that it is possible to distinguish between receptor and non-receptor tyrosine kinase activities by Raman spectroscopy and imaging.

The localization of receptors with tyrosine kinase activity on lipid droplets reveals the unexpected role of lipid droplets in cancer cells. The results presented in this study suggest that lipid droplets are functional organelles that are not only responsible for fat storage, but are also involved in lipid metabolism with enhanced phosphorylation of proteins, as well as participating in cancer development.

We proposed a mechanism of molecular processes that explains the interplay between glucose metabolism in cancer, increased de novo lipid synthesis and amplified tyrosine kinase phosphorylation activity.

To summarize, we think that separately, cancer biology with genomics and proteomics protocols provides only a partial picture of cancer, and we hope that an interdisciplinary approach—utilizing conventional biology tools and Raman tools—is greater than the sum of its parts to attain a better, in-depth mechanistic connection of phosphorylation to the molecular basis of cancer.

## Figures and Tables

**Table 1:** Raman intensity ratios, I_1243_/I_1263_; I_1586_/I_829_; I_1586_/I_1004_; and I_1586_/I_1658_, for normal and cancerous breast (invasive ductal carcinoma G3), small intestine (adenocarcinoma G2) and tumorous of brain (medulloblastoma) tissue samples. N – normal tissue, C – cancerous/tumorous tissue, SD – standard deviation, *p* – probability value.

Ratio	Assignment	Type	Breast		Small Intestine		Brain	
			Mean ± SD	*p*	Mean ± SD	*p*	Mean ± SD	*p*
1243/1263	phosphorylated proteins/non-phosphorylated proteins Amide III	N	0.86 ± 0.18	0.51	0.95 ± 0.20	0.41	0.76 ± 0.04	0.03
		C	0.99 ± 0.25		1.06 ± 0.05		1.59 ± 0.44	
1586/829	phosphorylated proteins/tyrosine	N	0.74 ± 0.42	0.02	1.93 ± 0.67	0.01	2.01 ± 0.68	0.04
		C	2.35 ± 0.63		4.62 ± 0.68		11.12 ± 5.02	
1586/1004	phosphorylated proteins/ phenyloalanine	N	0.27 ± 0.10	0.08	1.13 ± 0.21	0.02	0.81 ± 0.45	0.03
		C	0.69 ± 0.30		1.60 ± 0.10		3.49 ± 1.27	
1586/1658	phosphorylated proteins/proteins	N	0.19 ± 0.06	0.15	0.41 ± 0.19	0.15	0.34 ± 0.05	0.05
		C	0.31 ± 0.10		0.61 ± 0.05		3.03 ± 1.71	

**Table 2:** Partial least squares discriminant analysis (PLSDA) with receiver operating characteristic area under the curve (ROC AUC) of brain, small intestine and breast tissues.

Parameter	Brain		Small Intestine		Breast	
	Tumor	Normal	Cancer	Normal	Cancer	Normal
Calibration						
Sensitivity	100.0%	100.0%	100.0%	100.0%	66.7%	100.0%
Specificity	100.0%	100.0%	100.0%	100.0%	100.0%	66.7%
AUC	1.00	1.00	1.00	1.00	0.89	0.89
Cross validation						
Sensitivity	100.0%	100.0%	66.7%	66.7%	66.7%	66.7%
Specificity	100.0%	100.0%	66.7%	66.7%	66.7%	66.7%
AUC	1.00	1.00	0.89	0.89	0.78	0.78

**Table 3:** Raman intensity ratios, I_1243_/I_1263_; I_1586_/I_829_; I_1586_/I_1004_; and I_1586_/I_1658_, for normal human astrocytes (NHA, N) and glioblastoma (U87 MG, U) cell lines, SD – standard deviation, *p* – probability value.

Ratio	Assignment	Cell	Lipid Droplets		Mitochondria		Nucleus		Cytoplasm	
			Mean ± SD	*p*	Mean ± SD	*p*	Mean ± SD	*p*	Mean ± SD	*p*
1243/1263	phosphorylated proteins/non-phosphorylated proteins Amide III	N	1.18 ± 0.19	0.03	1.10 ± 0.26	0.38	1.34 ± 0.19	0.04	1.01 ± 0.28	0.31
		U	0.70 ± 0.15		0.91 ± 0.21		0.92 ± 0.16		0.82 ± 0.06	
1586/829	phosphorylated proteins/tyrosine	N	4.49 ± 0.44	0.12	3.98 ± 0.53	0.23	3.13 ± 0.53	0.15	2.71 ± 0.28	0.67
		U	3.46 ± 0.81		3.45 ± 0.37		2.20 ± 0.75		2.94 ± 0.83	
1586/1004	phosphorylated proteins/phenyloalanine	N	1.78 ± 0.29	0.23	2.21 ± 0.56	0.07	1.37 ± 0.15	0.81	1.91 ± 0.52	0.78
		U	1.54 ± 0.02		1.37 ± 0.23		1.34 ± 0.14		1.79 ± 0.45	
1586/1658	phosphorylated proteins /proteins	N	0.59 ± 0.17	0.40	0.69 ± 0.08	0.01	0.50 ± 0.03	0.76	0.54 ± 0.08	0.04
		U	0.47 ± 0.14		0.46 ± 0.04		0.52 ± 0.10		0.40 ± 0.00	
